# Retzus-sparing robotic-assisted laparoscopic radical prostatectomy: a step–by-step technique description of this first brazilian experience

**DOI:** 10.1590/S1677-5538.IBJU.2015.0348

**Published:** 2016

**Authors:** Marcos Tobias-Machado, Igor Nunes-Silva, Alexandre Kiyoshi Hidaka, Leticia Lumy Kanawa Sato, Roberto Almeida, Jose Roberto Colombo, Hamilton de Campos Zampolli, Antonio Carlos Lima Pompeo

**Affiliations:** 1 Departamento de Urologia, Faculdade de Medicina do ABC, Santo Andre, SP, Brasil; 2 Departamento de Uro-Oncologia, Instituto do Câncer do Estado de São Paulo, São Paulo, SP, Brasil; 3 Departamento de Urologia, Faculdade de Medicina, Universidade de São Paulo, São Paulo, SP, Brasil

## Abstract

**Introduction:**

Retzus-sparing robotic-assisted radical prostatectomy(RARP) is a newly approach that preserve the Retzus structures and provide better recovery of continence and erectile function. In Brazil, this approach has not yet been previously reported.

**Objective:**

Our goal is to describe Step-by-Step the Retzus-sparing RARP surgical technique and report our first Brazilian experience.

**Methods:**

We present a case of a 60-year-old white man with low risk prostate cancer. Surgical materials were four arms Da Vinci robotic platform system, six transperitoneal portals, two prolene wires and Polymer Clips. This surgical technique was step-by-step described according to Galfano et al. One additional step was added as a modification of Galfano et al. Primary technique description: The closure of the Denovellier fascia.

**Results:**

We have operated one patient with this technique. The operative time was 180minutes, console time was135 min, the blood loss was 150ml, none perioperative or postoperative complications was found, hospital stay of 01 day. The anatomopathological classification revealed a pT2aN0M0 specimen with free surgical margins. The patient achieved continence immediately after bladder stent retrieval. Full erection reported after 30 days of surgery.

**Conclusion:**

Retzus-sparing RARP approach is feasible and reproducible. However, further comparative studies are necessary to demonstrate potential benefits in continence and sexual outcomes over the standard approaches.

